# The Promoting Role of Teachers’ Emotional Competence in Innovative Teaching Behaviors: The Mediating Effects of Teaching Efficacy and Work Vitality

**DOI:** 10.3390/bs15101357

**Published:** 2025-10-05

**Authors:** Xi Li, Si Cheng, Ning Chen, Haibin Wang

**Affiliations:** 1 School of Psychology, Shanghai Normal University, Shanghai 200234, China; 2 College of Educational Science, Huangshan University, Huangshan 245041, China

**Keywords:** teachers’ emotional competence, innovative teaching behaviors, teaching efficacy, work vitality, job demands–resources theory

## Abstract

Amid ongoing educational reforms and the rapid advancement of the knowledge economy, innovative teaching behaviors are not only closely related to teachers’ professional growth and students’ academic achievement but are also regarded as the key driving force for the evolution of the educational system. Consequently, identifying effective ways to foster teachers’ innovative teaching behaviors has become a central concern in educational psychology and management. Grounded in the Job Demands–Resources framework, this study developed and tested a chained mediation model using survey data from 1165 Chinese elementary and secondary school teachers. The model examines how teachers’ emotional competence fosters innovative teaching behaviors and elucidates the underlying mechanisms. The results revealed that (1) emotional competence significantly and positively predicted innovative teaching behaviors, and (2) teaching efficacy and work vitality served not only as independent mediators but also as sequential mediators in this relationship. These findings extend the understanding of the antecedents of teachers’ innovative behaviors from an emotional perspective, demonstrating that emotional competence, as a critical psychological resource, can be transformed into innovative teaching behaviors through dual “cognitive–motivational” and “energy–motivational” pathways. This study offers both theoretical insights and practical implications for advancing teaching innovation by strengthening teachers’ emotional competence, teaching efficacy, and work vitality.

## 1. Introduction

Innovative teaching behavior refers to teachers’ engagement in innovation within the school teaching context, encompassing the deliberate generation, implementation, modification, and dissemination of relatively novel instructional ideas or methods aimed at enhancing teaching effectiveness (Liu et al., 2024). Amid current educational reforms and the rapid advancement of the knowledge economy, teachers’ innovative teaching behaviors have become not only a vital means of enhancing students’ academic achievement, but also a key force in driving continuous school improvement and a solution to the complex challenges of contemporary education (OECD, 2025). Consequently, identifying effective strategies to foster teachers’ innovative teaching behaviors has become a central concern in educational psychology and management. While prior research has predominantly examined the predictive roles of organizational factors and individual cognitive resources in shaping innovative work behaviors (Nguyen et al., 2022; Yang et al., 2025), the affective dimensions of teachers’ innovation processes have received comparatively little attention. Teaching is not solely a cognitive process but also a profoundly emotional activity that requires teachers to perceive, interpret, and regulate both their own and their students’ emotions (Hargreaves, 2001). Emotional competence—a multifaceted quality encompassing abilities such as emotional awareness, contagion, and regulation (Mayer et al., 2016; E. Savina et al., 2025)—is expected to shape innovative teaching behaviors. However, empirical and theoretical investigations of how emotional competence translates into innovative teaching behaviors remain limited, particularly regarding the specific mechanisms underlying this relationship. Addressing this gap, the present study investigates the role of teachers’ emotional competence in fostering innovative teaching behaviors and its underlying mechanisms. By doing so, it enriches the theoretical framework of educational psychology and teacher professional development while offering practical insights for teacher training and school innovation in the context of ongoing educational reform.

## 2. Theoretical Framework and Literature Review

### 2.1. Theoretical Framework

The Job Demands–Resources (JD-R) model is a widely influential framework in occupational health psychology (Bakker & Demerouti, 2017; Bakker et al., 2023). At its core, the model proposes a parsimonious dual structure: despite the diversity of job characteristics, they can be categorized into two fundamental dimensions—job demands and job resources. Job demands are aspects of work that require sustained physical, cognitive, or emotional effort, whereas job resources are aspects that facilitate goal attainment, buffer the negative impact of demands, and foster development (Demerouti et al., 2001). According to JD-R, the dynamic balance between these two dimensions initiates opposing processes. When resources exceed demands, the gain process is activated, generating sustained motivation and enhancing work engagement. Conversely, when demands surpass resources, the strain process is triggered, depleting energy reserves and contributing to burnout symptoms such as emotional exhaustion and physical fatigue (Bakker & Demerouti, 2017).

Teaching is inherently emotionally intensive, requiring teachers to perceive, interpret, and regulate both their own emotions and those of their students to optimize instructional outcomes (Hargreaves, 2001). This places high demands on teachers’ emotional competence. Emotional competence—an integrated quality encompassing emotional awareness, contagion, and regulation—helps educators meet these emotional requirements (E. Savina et al., 2025). From a JD-R perspective, emotional competence constitutes a core personal resource that supports teachers in fulfilling the emotional demands of their profession.

Furthermore, drawing on the JD-R model’s gain pathways, this study posits that work motivation, stimulated by abundant personal resources (emotional competence) functions as a driving force for positive outcomes, including innovative teaching behaviors. Thus, the JD-R model demonstrates strong applicability to this research: it not only offers a meta-theoretical foundation for situating emotional competence as a key resource but also provides a clear framework for examining the motivational mechanisms through which emotional competence fosters innovation in teaching. This framework anchors the investigation of how teachers’ emotional competence promotes innovative teaching behaviors within the robust theoretical tradition of occupational health psychology.

### 2.2. The Promoting Role of Teachers’ Emotional Competence on Innovative Teaching Behaviors

Amid the global transformation of educational paradigms, the increasing diversity of student populations, the rapid advancement of educational technologies, and rising societal expectations for educational effectiveness collectively demand that teachers adopt more innovative teaching behaviors. These behaviors are defined as the development and application of novel, original, or creative instructional methods aimed at improving teaching outcomes (Liu et al., 2024). Conceptually, while innovative teaching behaviors overlap with teaching creativity, they differ in scope: teaching creativity emphasizes the generation of new ideas, whereas innovative teaching behaviors encompass both the generation and implementation of those ideas in practice (Anderson et al., 2014). Empirical evidence indicated that innovative teaching behaviors yield a range of positive outcomes, including higher student learning satisfaction (Suyudi et al., 2022), stronger intrinsic goal orientation (Maun et al., 2023), and improved academic performance (Schacter et al., 2006).

Research on the antecedents of innovative teaching behaviors has primarily focused on two domains: the organizational environment, such as leadership styles and institutional support (Nguyen et al., 2022), and individual cognitive attributes, such as self-efficacy (Pazin et al., 2022). However, given the affective nature of teaching (Hargreaves, 2001; E. Savina et al., 2025), innovative teaching behaviors are influenced not only by the structural aspects of the educational environment or individual cognitive processes but also by teachers’ capacity to recognize, regulate, and mobilize emotions. This emotional competence may significantly shape their ability to engage in innovative teaching practices (Jiang & Tong, 2025). Despite this, the role of emotional factors in teachers’ innovative processes has been relatively neglected.

In this study, teachers’ capacity to regulate emotions is conceptualized as emotional competence. Despite its growing significance, no unified consensus has been reached in academic circles regarding the scope of teachers’ emotional competence, with multiple classification approaches proposed. For example, Mayer et al. (2016) introduced a competency model of emotional intelligence comprising four branches: recognition, utilization, comprehension, and regulation. Bar-On (2006) advanced a five-factor model grounded in the social–emotional intelligence framework, while E. Savina et al. (2025) identified eight discrete emotional skills. Collectively, these studies indicate that emotional competence is neither rigid nor unidimensional but can be functionally categorized according to research focus and contextual demands. Building on this foundation, the present study defines teachers’ emotional competence as the capacity to perceive, influence, and regulate both their own and their students’ emotions during teaching. From a process perspective, emotional awareness serves as the entry point, enabling teachers to accurately identify and interpret emotional states in themselves and their students, thereby providing critical insights into the classroom’s emotional climate (Mayer et al., 2016; Brackett et al., 2011). Following awareness, emotional contagion occurs as teachers’ emotions are both influenced by students and transmitted back through teachers’ emotional expressions, creating a bidirectional dynamic. This resonance can amplify positive emotions and foster engagement or, under negative conditions, diminish students’ motivation to learn (Frenzel et al., 2018). Ultimately, emotional regulation enables teachers to manage and guide this process: regulating their own responses to mitigate burnout and ineffectiveness caused by negative contagion, while also fostering positive atmospheres that help students overcome adverse emotions and achieve instructional goals (Gross, 2015; Jennings & Greenberg, 2009).

In summary, emotional awareness provides informational input, emotional contagion reflects interaction and transmission, and emotional regulation ensures control and transformation. Together, these three components constitute a coherent, process-oriented mechanism through which teachers’ emotional competence is transformed into psychological resources that enhance learning and drive innovative teaching in high-emotional-labor contexts.

Empirical evidence supports these claims: high levels of emotional competence are positively associated with teaching effectiveness, stronger teacher–student relationships, and greater teacher well-being (E. Savina et al., 2025). Emotional competence is also regarded as a prerequisite for effective teaching (Vignjević Korotaj & Mrnjaus, 2021). While direct evidence linking teachers’ emotional competence to innovative teaching behaviors is limited, theoretical perspectives provide a strong rationale for such a relationship. First, according to the broaden-and-build theory of positive emotions, positive affect enhances cognitive flexibility and fosters expansive thinking (Fredrickson, 2001). Teachers with high emotional competence are more likely to experience positive emotions in their daily practice (Parke et al., 2015), which in turn cultivates creativity and facilitates the enactment of innovative teaching strategies (Jaussi et al., 2017). Second, from the JD-R perspective, high emotional competence functions as a critical job resource, enabling teachers to effectively regulate emotional expenditure—a core job demand—during instructional interactions. In doing so, it transforms potential emotional challenges into opportunities to cultivate positive teacher-student relationships (E. Savina et al., 2025) and fulfills the emotional competency requirements inherent in teaching (Demerouti et al., 2001). By reducing the cognitive load associated with basic emotional coping, emotional competence allows teachers to redirect their mental resources toward complex and creative pedagogical explorations, thereby fostering the emergence of innovative teaching behaviors. Beyond theoretical reasoning, empirical findings from non-teaching populations also support the notion that affective competence positively predicts innovative behaviors (Darvishmotevali et al., 2018; Stawicki et al., 2023). In summary, this study hypothesizes that (H1) teacher emotional competence may serve as a resource-based antecedent factor that facilitates the emergence of innovative teaching behaviors.

### 2.3. Independent Mediating Roles of Teaching Efficacy and Work Vitality

The preceding discussion highlights the facilitating role of teachers’ emotional competence in innovative teaching behaviors; however, the underlying mechanisms through which emotional competence exerts its influence remain to be further clarified (Liu et al., 2024). Drawing on the JD-R model’s gain pathway, this study posits that teachers’ affective competence, as a critical job resource, can stimulate intrinsic motivation when aligned with the emotional demands of teaching tasks, thereby driving innovative teaching behaviors as a key manifestation of work engagement (Bakker et al., 2023; Demerouti et al., 2001).

Teaching efficacy as a motivational mediator. As a core psychological resource, teaching efficacy governs teachers’ goal setting, effort direction, and persistence in instructional tasks (Bandura, 1977). Fundamentally, it reflects teachers’ confidence in their ability to implement instruction successfully and achieve educational goals (Tschannen-Moran & Hoy, 2001). Empirical evidence consistently demonstrated a strong positive association between teachers’ emotional competence and their sense of efficacy (Chen et al., 2024; Kyriazopoulou et al., 2025). On one hand, high emotional competence enables teachers to sustain positive emotions in daily teaching (E. Savina et al., 2025), thereby providing the affective arousal necessary for efficacy development (Bandura, 1977). On the other hand, emotional competence can enhance instructional effectiveness (Garner, 2010), offering direct mastery experiences that further consolidate teachers’ efficacy beliefs (Bandura, 1977). Compared with other intrinsic motivational factors, teaching self-efficacy more directly reflects teachers’ assessments of their own capabilities and beliefs within innovative contexts, explaining their willingness to persistently adopt new methods. Among the various individual and organizational factors influencing teachers’ innovative behaviors, self-efficacy has been identified as the dominant motivational driver (Pazin et al., 2022; Thurlings et al., 2015). Drawing on social cognitive theory (Bandura, 1977), the mechanism lies in the belief that confidence in achieving innovative outcomes strengthens motivation, persistence, and resilience in the face of challenges (Bandura & Locke, 2003). Integrating this evidence, the study proposes that teaching efficacy, a key psychological resource derived from emotional competence, effectively stimulates innovation motivation and fosters persistence in innovation. Accordingly, this study hypothesizes that (H2) teaching efficacy mediates the relationship between emotional competence and innovative teaching behaviors.

Work vitality serves as a motivational mediator. Unlike the belief-oriented nature of teaching efficacy, work vitality reflects the energy and enthusiasm that teachers invest in their work—an energized psychological state that arises when work resources are adequately met (Ryan & Deci, 2008). Work vitality reflects a sustained positive emotional response during ongoing interactions with the work environment (Shirom, 2011) and has been shown to be influenced by employees’ emotional competence (Carmeli et al., 2014; Weiher et al., 2022). For teachers, high emotional competence provides effective emotion regulation strategies that mitigate the impact of stress and negative emotions such as anxiety or frustration in high-pressure teaching environments, thereby conserving psychological energy and preventing emotional exhaustion (Jennings & Greenberg, 2009). Simultaneously, emotionally competent teachers, equipped with interpersonal communication and conflict management skills, are better able to cultivate positive relationships with students, colleagues, and parents (Poulou & Garner, 2025), reducing socioemotional burnout and sustaining vitality (Mérida-López & Extremera, 2017). In terms of outcomes, work vitality differs from broader constructs such as overall work engagement or job satisfaction. It captures a sense of forward momentum, emphasizing teachers’ immediate energy levels and drive during specific teaching activities (Lavrusheva, 2020). This momentum not only supports the completion of daily tasks but also motivates teachers to proactively explore new technologies, methods, and practices, thereby enhancing the likelihood of innovative improvements in teaching performance (Op den Kamp et al., 2020; Kark & Carmeli, 2009). Thus, work vitality is not merely a byproduct of resource fulfillment but serves as the energetic foundation sustaining innovative behavior (Shirom, 2011)—a crucial “energy–motivation” link connecting emotional competence and innovative teaching. Consequently, this study hypothesizes that (H3) work vitality mediates the relationship between emotional competence and innovative teaching behaviors.

### 2.4. Chain-Mediated Effect of Teaching Efficacy and Work Vitality

The preceding discussion has outlined the independent motivational mediating roles of teaching efficacy and work vitality in the relationship between teachers’ emotional competence and innovative teaching behaviors. However, the dynamic interplay between these two motivational factors requires further clarification. From the perspective of self-determination theory, work vitality can be understood as a product of the satisfaction of basic psychological needs (Ryan & Deci, 2008). Specifically, the satisfaction of relatedness, competence, and autonomy needs determines the extent to which teachers experience vitality in their work (Karkkola et al., 2019). Among these, competence needs refer to the desire to experience effective control and mastery in interactions with the environment (Ryan & Deci, 2008). Teaching efficacy, defined as teachers’ confidence in their ability to achieve pedagogical goals (Tschannen-Moran & Hoy, 2001), reflects this sense of mastery. In the present study, teaching efficacy—nurtured by high levels of emotional competence—contributes to the satisfaction of competence needs, which in turn sustains teachers’ work vitality.

This suggests a motivational transfer effect between teaching efficacy and work vitality, positioning them as sequential mediators through which emotional competence influences innovative teaching behaviors. Teachers with strong efficacy beliefs are more likely to view teaching as a “demonstration of competence” rather than a “test of competence.” This cognitive reappraisal transforms teaching tasks into self-endorsed goals, thereby fostering stronger intrinsic motivation (Deci & Ryan, 2000). Such intrinsic motivation generates heightened energy and positive affect, which serve as reservoirs of sustainable psychological resources that invigorate teaching and learning (Nix et al., 1999) and drive innovative practices. This process constitutes a motivational transformation mechanism that can be summarized as a chain reaction of “competence conviction—vitality emergence” (Parker et al., 2010). Consequently, a chain-mediated pathway is formed whereby emotional competence promotes innovative teaching behaviors through teaching efficacy (as the primary motivational mediator) and work vitality (as the secondary motivational mediator). Building on this reasoning, the study proposes hypothesizes that (H4) teaching efficacy and work vitality jointly exert a chain mediating effect in the relationship between emotional competence and innovative teaching behaviors.

### 2.5. Current Research

As a central driver of educational reform, innovative teaching behaviors not only promote teachers’ professional development and enhance students’ academic achievement but also play a crucial role in the evolution of educational systems (OECD, 2025). However, teachers do not consistently engage in innovative teaching practices and may even demonstrate tendencies to avoid innovation (Sánchez & Gutiérrez-Esteban, 2023; N. N. Savina, 2019). This reluctance partly arises from the inherent risks of innovation, such as potential loss of classroom control, resistance or critique from parents and colleagues, and uncertainty about outcomes (Cheng, 2010; Jones & Le Fevre, 2021). Under such conditions of risk and ambiguity, the implementation of innovative teaching behaviors depends heavily on the availability of sufficient resources and strong intrinsic motivational drives that can counterbalance risk-averse tendencies (Gorozidis & Papaioannou, 2016; Kottmann et al., 2024).

Anchored in the JD-R framework (Bakker et al., 2023), this study investigates the role of internal motivation as a mechanism linking teachers’ emotional competence—a key psychological resource aligned with the affective demands of teaching (E. Savina et al., 2025)—to innovative teaching behaviors. Specifically, two motivational mechanisms are emphasized: teaching efficacy and work vitality. Teaching efficacy, shaped by the positive emotional arousal and mastery experiences associated with high levels of emotional competence (Bandura, 1977), enhances teachers’ confidence, persistence, and commitment to instructional innovation (Bandura & Locke, 2003). Work vitality, sustained through positive emotions and harmonious interpersonal relationships fostered by emotional competence (Mérida-López & Extremera, 2017; Poulou & Garner, 2025), energizes teachers to use innovation as a pathway for self-improvement and professional growth (Lavrusheva, 2020). Furthermore, teaching efficacy contributes to the internalization of instructional tasks as intrinsic goals (Deci & Ryan, 2000), which in turn fuels vitality (Nix et al., 1999), jointly propelling innovative teaching behaviors. Based on this, the present study identifies emotional competence as an antecedent predictor of innovative teaching behaviors, particularly under the premise of teaching innovation as a high-risk endeavor. It proposes three indirect process pathways: (a) an independent mediating path of teaching efficacy, (b) an independent mediating path of work vitality, and (c) a sequential chain-mediating path of teaching efficacy leading to work vitality.

**Figure 1:**
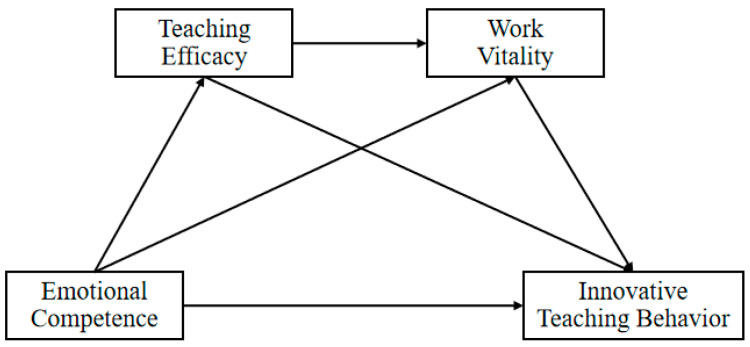
The hypothesized structural model illustrating the proposed direct and indirect relationships among emotional competence, teaching efficacy, work vitality, and innovative teaching behavior.

To empirically test these assumptions, this study employed a chain-mediated model using large-scale survey data on teachers’ emotional competence, teaching efficacy, work vitality, and innovative teaching behaviors. The hypothetical model is presented in Figure 1.

## 3. Method

### 3.1. Participants

A cluster sampling method was used to survey 1420 basic education teachers from public schools in China. After excluding participants who failed the lie detection check—which required selecting a specific response phrased as, “To ensure you have carefully read the question, please select ‘completely disagree’ for this item”—a total of 1165 valid questionnaires were retained, yielding an effective response rate of 82.04%. The mean age of the participants was Mage = 39.92 years (SDage = 11.26), of which 360 (30.90%) were males and 805 (69.10%) were females; 259 (22.23%) were kindergarten teachers, 580 (49.79%) were elementary school teachers, and 326 (27.98%) were secondary school teachers; 407 (34.94%) teachers had second grade title, 451 (38.71%) teachers had first grade title, and 133 (11.42%) teachers had senior grade title; 164 (14.08%) teachers were in urban areas, 410 (35.19%) teachers were in counties, 384 (32.96%) teachers were in towns, and 207 (17.77%) teachers were in villages. Each survey was conducted by trained graduate students in psychology. The study was approved by the Scientific Research Ethics Committee of the corresponding author’s institution.

### 3.2. Measures

#### 3.2.1. Emotional Competence

Building on the preceding analysis of teachers’ emotional competence and existing research (Alemdar & Anılan, 2020; Bar-On, 2006; Frenzel et al., 2018; Mayer et al., 2016; E. Savina et al., 2025), this study identifies emotional awareness (perceiving one’s own and students’ emotions), emotional contagion (sensing and transmitting emotions during teacher–student interactions), and emotional regulation (managing one’s own and students’ emotions through targeted strategies) as the core components of teachers’ emotional competence in daily teaching practice. The scale includes three dimensions of emotional awareness, emotional contagion and emotional regulation, with 14 items (Sample items are presented in Appendix A). These items were extracted and adapted from the Emotional Contagion Scale (Doherty, 1997), the Emotional Regulation Strategies Scale (Gross & John, 2003), and other maturation scales, using a 7-point Likert scale (from 1 = “not at all consistent” to 7 = “fully consistent”). The Cronbach’s alpha internal consistency coefficient for the Teachers’ Emotional Competence Scale in the current sample was 0.95. Confirmatory factor analysis indicated a good model fit (RMSEA = 0.07, CFI = 0.92, SRMR = 0.03), and the factor loadings for each item were approximately 0.80, demonstrating that the scale is reliable and valid for measuring teacher emotional competence.

#### 3.2.2. Teaching Efficacy

The Individual Teaching Efficacy Scale developed by Yu et al. (1995) was used to measure participants’ sense of teaching efficacy. The questionnaire consisted of 17 items (Sample items are presented in Appendix A) and was rated on a 7-point Likert scale (from 1 = “not at all consistent” to 7 = “fully consistent”). The Cronbach’s alpha internal consistency coefficient for the Individual Teaching Efficacy Scale for the current sample was 0.87. Confirmatory factor analysis showed that the model fit was acceptable (RMSEA = 0.07, CFI = 0.90, SRMR = 0.07), indicating that the scale is reliable and valid for measuring teaching efficacy.

#### 3.2.3. Work Vitality

The Work Vitality subscale of the Thriving Scale developed by Porath et al. (2012) was used to assess participants’ work vitality. The scale consists of five items (Sample items are presented in Appendix A), using a 7-point Likert scale (from 1 = “not at all consistent” to 7 = “fully consistent”). The Cronbach’s alpha internal consistency coefficient for the Work Vitality Scale for the current sample was 0.72. Confirmatory factor analysis showed that the model fit was good (RMSEA = 0.06, CFI = 0.97, SRMR = 0.02), indicating that the scale is reliable and valid for measuring work vitality.

#### 3.2.4. Innovative Teaching Behavior

The Teacher’s Innovative Work Behavior Scale developed by Zhang and Zhang (2012) was adopted to assess participants’ innovative behavior in teaching. The scale consists of three dimensions: innovative results, innovative activities, and willingness to innovate, with a total of 16 items (Sample items are presented in Appendix A), using a 7-point Likert scale (from 1 = “not at all consistent” to 7 = “fully consistent”). The Cronbach’s alpha internal consistency coefficient for the Teacher Innovative Work Behavior Scale for the current sample was 0.98. Confirmatory factor analysis showed that the model fit was good (RMSEA = 0.06, CFI = 0.94, SRMR = 0.03), indicating that the scale is reliable and valid for measuring innovative teaching behavior.

### 3.3. Data Processing and Analysis Methods

SPSS version 26 was used for data management and descriptive analysis, and all other analyses were done using Mplus software version 8. In order to explore the predictive role of teachers’ emotional competence on innovative teaching behavior and its process mechanism, a chain-mediated model was established based on resource preservation theory, in which teachers’ emotional competence was taken as the independent variable, innovative teaching behavior was taken as the dependent variable, and teaching efficacy and work vigor were taken as the sequential mediating variables, respectively. Specifically, the model includes one direct effect and three indirect effects, namely, the direct predictive effect of teachers’ emotional competence on innovative teaching behaviors, the independent mediating effect of teaching efficacy and job vitality, and the chain mediating effect of “teaching efficacy–job vitality” (the model is shown in Figure 1). In the above analysis, maximum likelihood estimation was used for parameter testing, Bootstrap method was used for confidence interval estimation, and participants’ gender, title, teaching level, and school location were used as control variables.

### 3.4. Common Method Bias

Since this study took a self-reported approach to data collection by the subjects, it was necessary to test for common method bias. The one-way CFA method was used to test for common method bias and the results showed a poor one-way model fit (RMSEA = 0.13 > 0.10, CFI = 0.63 < 0.90, SRMR = 0.12 > 0.10), which suggests that there was no significant common method bias in this study.

## 4. Results

### 4.1. Preliminary Analysis

The means, standard deviations and correlation coefficients of all variables at each time point are shown in Table 1. The results show that all variables showed at least a significant moderate positive correlation (*r*s = 0.63 to 0.86, *p*s < 0.001), reflecting significant interrelationships and supporting the suitability of the data for subsequent path analysis.

### 4.2. Testing the Mediation Model

**Figure 2:**
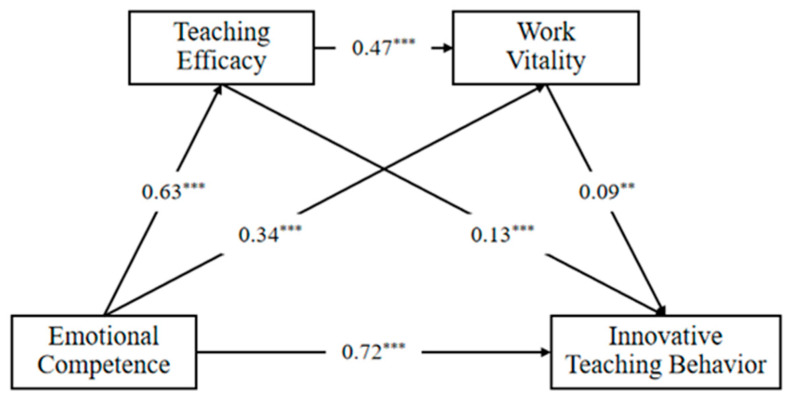
Direct and indirect effects of emotional competence on innovative teaching behavior through the mediating pathways of teaching efficacy and work vitality. Note: All betas are standardized path coefficients. ** *p* < 0.01, *** *p* < 0.001.

A chain-mediated model was used to test the predictive role of teachers’ emotional competence on innovative teaching behaviors and its process mechanisms. Model fit test indicated a saturated model: RMSEA = 0.00, CFI = 1.00, SRMR = 0.00. Detailed results are shown in Figure 2: Teachers’ emotional competence significantly and positively predicted teaching efficacy (β = 0.63, *p <* 0.001, 95%CI [0.58, 0.66]), work vitality (β = 0.34, *p <* 0.001, 95%CI [0.28, 0.39]), and innovative teaching behaviors (β = 0.72, *p <* 0.001, 95%CI [0.65, 0.79]). Teaching efficacy significantly positively predicted work vitality (β = 0.47, *p <* 0.001, 95%CI [0.41, 0.53]) and innovative teaching behaviors (β = 0.13, *p =* 0.011, 95%CI [0.08, 0.18]). And work vitality significantly positively predicted innovative teaching behaviors (β = 0.09, *p =* 0.001, 95%CI [0.04, 0.15]).

The results of the analysis of the mediating effects are presented in Table 2: Teaching efficacy (β = 0.08, *p* < 0.001, 95%CI [0.05, 0.11]) and work vitality (β = 0.03, *p* = 0.001, 95%CI [0.01, 0.05]) not only played a significant and independent mediating role in the prediction of teachers’ emotional competence on innovative teaching behaviors but also played a significant chain mediating role (β = 0.03, *p* = 0.002, 95%CI [0.01, 0.04]).

## 5. Discussion

This study examined the promoting role of teachers’ emotional competence in innovative teaching behavior and the mediating functions of teaching efficacy and work vitality within the analytical framework of the Job Demands–Resources (JD-R) theory. The results showed that emotional competence significantly and positively predicted innovative teaching behaviors, and that teaching efficacy and work vitality not only exerted independent mediating effects but also formed a chain mediating pathway of “teaching efficacy–work vitality.”

### 5.1. The Promoting Role of Teachers’ Emotional Competence in Innovative Teaching Behaviors

The findings indicated that teachers’ emotional competence significantly and positively predicted innovative teaching behavior, suggesting that teachers with higher levels of emotional competence are more likely to engage in innovative practices. This result aligns with the studies of Parke et al. (2015) and Sung et al. (2020), which demonstrated that affective competence promotes creativity. Furthermore, it supports the Job Demands–Resources (JD-R) theory’s proposition that when individuals possess resources exceeding job demands, they are more likely to invest effort in proactive work-related behaviors, including teaching innovation (Bakker et al., 2023).

Teaching, by its very nature, is not only a cognitive but also a profoundly emotional practice, requiring teachers to perceive, understand, and regulate both their own and their students’ emotions in order to optimize teaching and learning outcomes (Hargreaves, 2001). Emotional competence, defined as an integrative capacity to perceive, regulate, and communicate emotions (E. Savina et al., 2025), can thus be conceptualized as a critical psychological resource for pedagogical innovation. This resource operates through two main mechanisms. First, the ability to generate and harness positive emotions expands teachers’ cognitive flexibility (Fredrickson, 2001), enabling them to identify instructional challenges more effectively and integrate cross-domain knowledge with greater openness, thereby fostering the creation and implementation of innovative solutions. Second, emotionally competent teachers are more inclined to adopt affective teaching strategies that enhance student engagement and learning (Kliueva & Tsagari, 2018). Compared with traditional cognition-centered strategies, affective strategies are inherently more adaptive and innovative, thereby directly contributing to the renewal of teaching practices.

### 5.2. Mediating Effects of Teaching Efficacy and Work Vitality

In addition to its direct effects, this study identified three indirect pathways through which teachers’ emotional competence fosters innovative teaching behaviors: the independent mediating role of teaching efficacy, the independent mediating role of work vitality, and the sequential chain-mediated of “teaching efficacy–work vitality.” These findings further support the motivational process hypothesis of the JD-R theory, which posits that when individuals’ resources sufficiently meet job demands, both intrinsic and extrinsic motivations are activated, driving goal-directed positive work behaviors (Bakker et al., 2023). These findings contrast with the mediating pathways reported by Jiang and Tong (2025) between psychological empowerment and career commitment under self-determination theory. Grounded in the JD-R framework, this study highlights the motivational roles of teaching efficacy and work engagement in the mechanism through which teachers’ emotional competence fosters innovative teaching behaviors.

Teaching efficacy, as a core mediator of the motivational gain pathway, reflects a positive belief in competence that emerges from affective resources, driving teachers toward innovation. Teachers with higher levels of emotional competence typically demonstrate stronger emotion regulation and interpersonal emotional communication skills, enabling them to evoke positive emotions more frequently and accumulate successful teaching experiences (E. Savina et al., 2025; Garner, 2010). Since positive affective arousal and mastery experiences are critical sources of teaching efficacy (Bandura, 1977), emotional competence facilitates the development of efficacy by cultivating these experiences. In turn, teaching efficacy significantly influences teachers’ goal setting, engagement, and resilience (Bandura, 1977). Teachers with strong efficacy beliefs tend to set more ambitious goals, demonstrate deeper cognitive and emotional involvement in instructional contexts, and sustain effort under challenging conditions (Tschannen-Moran & Hoy, 2001). Moreover, efficacy beliefs act as cognitive filters, guiding teachers to interpret challenges as opportunities for growth rather than threats (Bandura, 1977). Such positive appraisal patterns stabilize motivation, reinforce persistence, and ultimately enhance innovative teaching behavior. In short, when teachers believe in their capacity to achieve desired innovative outcomes, they are more motivated to act and persevere in realizing them.

Work vitality, another key mediator in the motivational gain pathway, represents a high-energy motivational state derived from the transformation of emotional resources into sustained psychological energy. Emotional competence enhances vitality by reducing surface acting, mitigating emotional exhaustion caused by labor-intensive regulation (Mérida-López & Extremera, 2017), while also strengthening positive affective experiences such as teaching enjoyment through effective emotion regulation. These positive states continuously replenish psychological energy reserves (Shirom, 2011). Vitality, as a motivational resource, is valuable not only because it provides the physical and psychological energy required for innovation (e.g., maintaining focus during complex instructional design) but also because it generates a sense of forward momentum and intrinsic enthusiasm (Lavrusheva, 2020). This energetic state amplifies teachers’ intrinsic motivation, encouraging them to set ambitious goals and engage their cognitive–emotional resources more fully (Ryan & Deci, 2008), thereby facilitating sustained engagement in innovative teaching practices.

In addition, the sequential mediation of “teaching efficacy–work vitality” highlights the recursive transmission mechanism between cognitive and energetic motivational resources. Teaching efficacy, as a central competence belief, fosters work vitality by catalyzing both the conservation and generation of energy. According to social cognitive theory, efficacy not only directly influences behavioral choices but also creates the conditions for vitality to emerge by shaping cognitive and affective appraisals (Bandura, 1977). Teachers with high efficacy are more likely to interpret instructional challenges as manageable rather than threatening (Judge & Bono, 2001), reducing stress-related energy depletion. At the same time, efficacy drives teachers to set more challenging goals and to invest more deeply in cognitive and affective resources (Tschannen-Moran & Hoy, 2001). Such engagement produces heightened experiences of meaning, mastery, and positive affective feedback (Rich et al., 2010), which serve as key psychological sources of vitality (Shirom, 2011). Therefore, teaching efficacy can effectively stimulate and sustain teachers’ work vitality through two complementary pathways: by reducing energy depletion and by promoting energy acquisition. In this process, the initial emotional resources derived from emotional competence are transformed into strong efficacy beliefs, which ultimately manifest as sustained teaching energy and continuous innovative momentum (Parker et al., 2010).

### 5.3. Contributions and Limitations

At the theoretical level, this study contributes in three key ways. First, it expands research on teachers’ emotional competence and innovative teaching behaviors. Prior studies have primarily focused on the effects of emotional competence on teacher well-being, classroom climate, or student emotions (Jennings & Greenberg, 2009; Brackett et al., 2011), and on organizational or cognitive factors influencing innovative teaching (Nguyen et al., 2022; Pazin et al., 2022). By linking emotional competence directly to innovative teaching behaviors, this study highlights its unique role in fostering pedagogical innovation. Second, it enriches the application of the Job Demands–Resources (JD-R) model in educational contexts, demonstrating that emotional competence, as a core personal resource, can stimulate positive teaching behaviors through the motivational pathway of teaching efficacy → work engagement, thereby validating and extending the JD-R model’s theoretical propositions (Bakker & Demerouti, 2017). Finally, the study uncovers a chain-mediated effect of teaching efficacy and work engagement, revealing that cognitive efficacy beliefs and energy-related psychological resources operate in a progressive manner to jointly propel innovative teaching behaviors, offering new perspectives and explanatory mechanisms for theoretical models of teacher innovation.

At the practical level, the findings provide actionable implications for teacher professional development and school management. First, in teacher training, both pre-service and in-service programs should go beyond conventional instructional skill training by systematically integrating emotional competence development. This can include courses or workshops focused on emotional awareness, regulation, and contagion. Through scenario simulations and reflective practice, these initiatives cultivate teachers’ ability to regulate emotions and positively influence students in high-pressure classrooms environments, thereby supplying essential psychological resources for innovative teaching. Second, at the school management level: educational administrators should foster an organizational climate that enhances teacher resources to promote classroom innovation. This includes establishing peer support mechanisms, cultivating a culture of positive feedback, and ensuring sufficient work resources, thereby providing both cognitive support (enhancing efficacy) and emotional support (boosting vitality) to facilitate experimentation with and sustained implementation of innovative practices. Finally, at the level of teacher self-development: educators can strengthen emotional competence through reflective practice and self-care, actively applying emotion regulation strategies in daily teaching to prevent burnout. Simultaneously, teachers should deliberately employ positive emotional expressions to inspire students and help them manage negative emotions during learning, further amplifying the momentum and effectiveness of innovative teaching.

Despite its contributions, this study has several limitations. First, the use of a cross-sectional design precludes firm causal inferences, even though the hypothesized relationships were supported by large-sample data. Bidirectional or dynamic interactions may exist among emotional competence, teaching efficacy, work vitality, and innovative teaching behavior. Future research could adopt longitudinal designs or experimental interventions to further clarify causal direction and temporal dynamics. Second, all variables were measured via teacher self-report. Although statistical tests suggested that common method bias was not a serious concern, the possibility of social desirability or recall bias remains. Future studies should incorporate multiple data sources, such as student evaluations, peer observations, or objective behavioral indicators (e.g., classroom video analyses, innovative lesson plan assessments), to enhance the validity and ecological robustness of the findings. Third, although this study features a substantial sample size, participants were drawn exclusively from partner schools affiliated with two regional universities in China. This resulted in an uneven distribution of teacher demographics and limited representativeness, potentially constraining the generalizability of the findings. Future research should broaden the sampling scope to enhance representativeness and strengthen the generalizability of the results. Finally, while this study established a chain mediation model, it did not account for organizational or contextual factors (e.g., school innovation climate, leadership support, policy environment) that may shape the link between emotional competence and innovative teaching. Subsequent studies could include such moderators to delineate the boundary conditions of the proposed mechanism.

## 6. Conclusions

In summary, this study demonstrated that teachers’ emotional competence significantly and positively predicted innovative teaching behavior. Furthermore, teaching efficacy and work vitality served not only as independent mediators but also as sequential mediators through the pathway of “teaching efficacy–work vitality.” These findings support the gain pathway proposed by the Job Demand–Resource model, extend the understanding of the antecedents of teachers’ innovative behaviors, and illuminate the role of emotional competence in fostering such behaviors along with its underlying mechanisms. Furthermore, they suggest that educational management strategies aimed at promoting teaching innovation should prioritize cultivating teachers’ emotional competence and enhancing their teaching efficacy and work vitality.

## Figures and Tables

**Table 1:** Means, standard deviations, and correlation coefficients of variables used in the hypothesized model.

	*M*	*SD*	1	2	3	4
1. Emotional competence	6.15	0.68	1			
2. Teaching efficacy	5.38	0.79	0.63 ***	1		
3. Work vitality	5.41	0.83	0.63 ***	0.68 ***	1	
4. Innovative teaching behavior	6.07	0.71	0.86 ***	0.66 ***	0.64 ***	1

Note: *** *p* < 0.001.

**Table 2:** Results of indirect effect testing.

Effect	β	*SE*	*p*	95%CI	Proportion
Total indirect effect	0.14	0.02	<0.001	[0.14, 0.24]	
Indirect effect 1	0.03	0.01	0.001	[0.01, 0.08]	22.06%
Indirect effect 2	0.08	0.02	<0.001	[0.06, 0.11]	58.82%
Indirect effect 3	0.03	0.01	0.002	[0.04, 0.09]	19.12%

Note: Indirect effect 1 represents the indirect path: “Emotional competence–Teaching efficacy–Innovative teaching behavior.” Indirect effect 2 represents the indirect path: “Emotional competence–Work vitality–Innovative teaching behavior.” Indirect effect 3 represents the indirect path: “Emotional competence–Teaching efficacy–Work vitality–Innovative teaching behavior.”.

## Data Availability

The data supporting the findings of this study are available from the corresponding author upon reasonable request.

## Appendix A

This appendix presents representative sample items from the translated versions of the primary scales used in this study (the original scales may include additional items).

1. *Sample Items from the Emotional Literacy Scale (7-point Likert scale):*

When a student receives praise, I genuinely share in their joy.

When a student feels down, I also experience discouragement and anxiety.

I pay special attention to students who face emotional challenges in class and seek appropriate ways to help them manage their emotions.

2. *Sample Items from the Teaching Efficacy Scale (7-point Likert scale):*

If a student in my class becomes disruptive, I am confident I can promptly correct their behavior.

If asked to teach a new subject, I believe I have the capability to do so.

3. *Sample Items from the Work Vitality Scale (7-point Likert scale):*

I feel energized and full of vitality in my work.

I approach my work with enthusiasm and vigor.

4. *Sample Items from the Innovative Teaching Practices Scale (7-point Likert scale):*

I strive to integrate innovative ideas into my teaching activities.

I flexibly employ diverse teaching methods to convey knowledge and skills.
